# Anti-Inflammatory Effect of IKK-Activated GSK-3β Inhibitory Peptide Prevented Nigrostriatal Neurodegeneration in the Rodent Model of Parkinson’s Disease

**DOI:** 10.3390/ijms23020998

**Published:** 2022-01-17

**Authors:** Seulah Lee, Dong Geun Hong, Seonguk Yang, Jaehoon Kim, Minwoo Baek, Seoyeong Kim, Dinakaran Thirumalai, Hae Young Chung, Seung-Cheol Chang, Jaewon Lee

**Affiliations:** 1 Department of Pharmacy, College of Pharmacy, Pusan National University, Busan 46241, Korea; leeseulah@pusan.ac.kr (S.L.); dgni21@naver.com (D.G.H.); ajy020603@gmail.com (S.Y.); wogns7799@naver.com (J.K.); qoralsdn1491@naver.com (M.B.); yamuns@naver.com (S.K.); hyjung@pusan.ac.kr (H.Y.C.); 2 Research Institute for Drug Development, Pusan National University, Busan 46241, Korea; 3 Department of Cogno-Mechatronics Engineering, College of Nanoscience and Nanotechnology, Pusan National University, Busan 46241, Korea; dinakaran@pusan.ac.kr (D.T.); s.c.chang@pusan.ac.kr (S.-C.C.)

**Keywords:** Parkinson’s disease, inhibitory κB kinase-activated GSK-3β inhibitory peptide, 1-methyl-4-phenyl-1,2,3,6-tetrahydropyridine, neuroinflammation, anti-inflammation

## Abstract

Parkinson’s disease (PD) is a progressive movement disorder caused by nigrostriatal neurodegeneration. Since chronically activated neuroinflammation accelerates neurodegeneration in PD, we considered that modulating chronic neuroinflammatory response might provide a novel therapeutic approach. Glycogen synthase kinase 3 (GSK-3) is a multifunctional serine/threonine protein kinase with two isoforms, GSK-3α and GSK-3β, and GSK-3β plays crucial roles in inflammatory response, which include microglial migration and peripheral immune cell activation. GSK-3β inhibitory peptide (IAGIP) is specifically activated by activated inhibitory kappa B kinase (IKK), and its therapeutic effects have been demonstrated in a mouse model of colitis. Here, we investigated whether the anti-inflammatory effects of IAGIP prevent neurodegeneration in the rodent model of PD. IAGIP significantly reduced MPP^+^-induced astrocyte activation and inflammatory response in primary astrocytes without affecting the phosphorylations of ERK or JNK. In addition, IAGIP inhibited LPS-induced cell migration and p65 activation in BV-2 microglial cells. In vivo study using an MPTP-induced mouse model of PD revealed that intravenous IAGIP effectively prevented motor dysfunction and nigrostriatal neurodegeneration. Our findings suggest that IAGIP has a curative potential in PD models and could offer new therapeutic possibilities for targeting PD.

## 1. Introduction

Parkinson’s disease (PD) is the second most common age-related neurodegenerative disease and is the result of dopaminergic neuron loss in the substantia nigra (SN) of the midbrain. PD is characterized clinically by bradykinesia; rigidity; trembling extremities; and nonmotor symptoms such as anxiety, sleep disorder, and dementia [1,2]. Since the etiology of PD is not completely understood, currently used clinical treatments such as levodopa (a dopamine precursor), pramipexole (a dopamine agonist), and monoamine oxidase B inhibitors target motor function control and the maintenance of endogenous dopamine levels, which are symptoms of PD [3]. However, these drugs have associated side effects such as dyskinesia and wearing-off. Dyskinesia causes involuntary muscle movements such as twitches, jerks, twisting, and writhing and is caused by high levels of PD drugs in the circulation. Wearing-off is a motor fluctuation that occurs between levodopa doses in ~80% of PD patients within 4 years of initiating levodopa therapy [4,5,6]. Furthermore, these drugs do not halt the progression of PD and require dosage increases to maintain their effects. Thus, rather than currently available drugs, which only attenuate clinical symptoms, a curative treatment is required to fully address PD.

Neuroinflammation maintains central nervous system (CNS) homeostasis; constitutes a reactive response against ischemia, infections, and injury; and is characterized by the activation of brain-specific immune cells (astrocytes and microglia) [7]. In pathologic conditions, chronically activated glial cells and infiltrating peripheral T cells accelerate neuroinflammation, which suggests that controlling neuroinflammation might provide a means of treating neurodegenerative diseases [8].

Glycogen synthase kinase 3 (GSK-3) was initially identified as an enzyme that controls glycogen metabolism, but it is now considered a key regulator of various cellular functions including inflammation and insulin signaling [9]. Furthermore, GSK-3β is known to be involved in neurogenesis [10], neuronal migration [11], and axon growth in the brain by controlling microtubule-associated proteins such as tau [12]. Moreover, as excessive GSK-3β activation contributes to tau pathology and amyloid beta synthesis, it is considered a therapeutic target in Alzheimer’s disease (AD). In addition, GSK-3β is known to promote microglial migration and proinflammatory mediator release. GSK-3β inhibitors were developed to treat neurodegenerative diseases such as AD, PD, bipolar disorder, and schizophrenia [13,14], but the long-term use of these inhibitors might disrupt many cellular processes because GSK-3β is a constitutively active kinase [15]. Therefore, it would be necessary to modulate the excessive activity of GSK-3β in response to pathological condition in neurodegenerative diseases without inhibiting physiological roles of GSK-3β.

In a previous study, GSK-3β inhibitory peptide was designed to be specifically activated by IKK (inhibitory kappa B kinase) [16]. IKK-activated GSK-3β inhibitory peptide (IAGIP) has a structure in which an IKK recognition motif and LRP6 motif are linked with serine. IKK recognition motif of IAGIP is phosphorylated when IKK activity is elevated, and the inserted serine is phosphorylated by GSK-3β, which transmits an IKK response to the LRP6 motif. Finally, the phosphorylated LRP6 motif directly inhibits GSK-3β. IAGIP has anti-inflammatory activity; for example, it increased anti-inflammatory interleukin (IL)-10 production and suppressed NF-κB activity in RAW264.7 and HCT116 cells in response to tumor necrosis factor (TNF) or lipopolysaccharide (LPS)-induced IKK activation. IAGIP also suppressed NF-κB-regulated protein levels and increased IL-10 levels in a 2,4,6-trinitrobenzene sulfonic acid-induced rat model of colitis [16].

However, it is unknown whether IAGIP has therapeutic effects in neurodegenerative diseases. In the present study, we investigated whether IAGIP suppresses glial activation and protects neurons in vitro and in a mouse model of PD.

## 2. Results

### 2.1. IAGIP Downregulated Astrocyte Activation and Inflammatory Response in Primary Astrocytes

**Figure 1:**
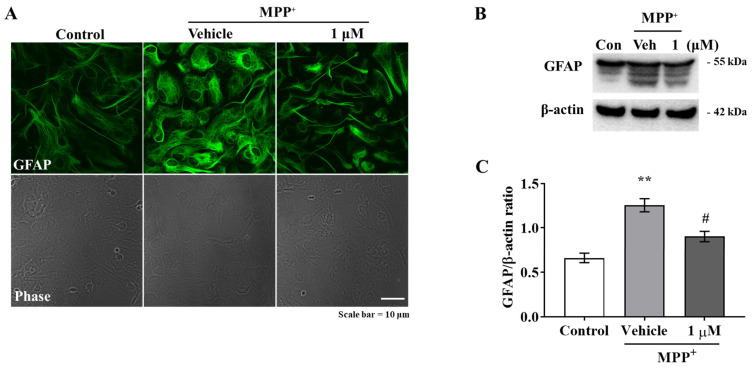

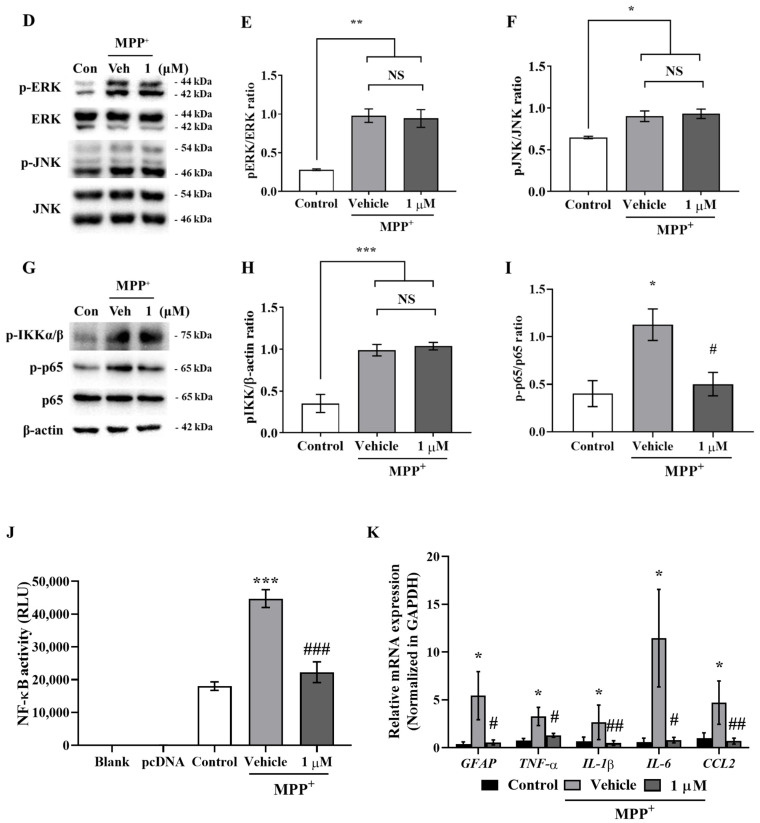
Anti-inflammatory effect of IAGIP on primary astrocytes. (**A**) Representative images showing that IAGIP attenuated GFAP fluorescence intensity (an astrocyte marker); scale bar = 10 μm. (**B**) Western blot analysis confirmed the inhibitory effect of IAGIP on MPP^+^-induced glial activation. (**C**) Western blot densitometry results. Three independent experiments were performed (*n* = 3). ^**^ *p* < 0.01 vs. vehicle-treated controls and ^#^ *p* < 0.05 vs. MPP^+^-treated controls. (**D**,**G**) Western blot showed that IAGIP reduced p65 phosphorylation without affecting MAPK activation. (**E**,**F**,**H**,**I**) Western blot densitometry results. Three independent experiments were performed (*n* = 3). ^***^ *p* <0.001, ^**^ *p* <0.01 and ^*^ *p* < 0.05 vs. vehicle-treated controls, and ^#^ *p* < 0.05 vs. MPP^+^-treated controls. (**J**) Relative luminescence units (RLU) were used to assess NF-κB promotor activity. Values are means ± SEs (*n* = 8). ^***^ *p* < 0.001 vs. vehicle-treated controls and ^###^ *p* < 0.001 vs. MPP^+^-treated controls. (**K**) RT-PCR showed that IAGIP reduced the MPP^+^-induced mRNA expressions of inflammatory cytokines and CCL2. Values are means ± SEs (*n* = 3–4). ^*^ *p* < 0.05 vs. vehicle-treated controls, and ^##^ *p* < 0.01 and ^#^ *p* < 0.05 vs. MPP^+^-treated controls.

Previous studies have demonstrated that MPP^+^ neurotoxin triggers glial activation and neuroinflammation by increasing levels of GFAP (glial fibrillary acidic protein) and proinflammatory cytokines in primary astrocytes [17,18,19]. We pre-treated primary astrocytes with 1 μM of IAGIP for 6 h and then co-treated them with 200 μM of MPP^+^ for 24 h. Immunocytochemistry (ICC) and Western blotting showed that IAGIP suppressed MPP^+^-induced GFAP increases in primary astrocytes (Figure 1A–C). It was previously reported that MPP^+^-induced glial activation involves MAPK activations [20]. However, although MPP^+^ increased the levels of phosphorylated ERK and JNK, IAGIP failed to attenuate the activations of ERK and JNK (Figure 1D–F). Interestingly, IAGIP significantly inhibited p65 phosphorylation (a subunit of NF-κB) without affecting IKK activation (Figure 1G–I). Luciferase assay confirmed that MPP^+^ increased the transcriptional activity of NF-κB and that IAGIP significantly suppressed the MPP^+^-induced promotor activity of NF-κB (Figure 1J). RT-PCR analysis revealed that MPP^+^ increased GFAP mRNA levels and that this was effectively reduced by IAGIP. Moreover, inflammatory cytokines such as TNF-α, IL-1β, IL-6, and chemokine CCL2, which are known downstream factors of NF-κB, were significantly increased by MPP^+^, and IAGIP significantly blocked these increases (Figure 1K). These results demonstrate that IAGIP effectively reduced MPP^+^-induced astrocyte activation and inflammatory response by inhibiting the NF-κB pathway in primary astrocytes.

### 2.2. IAGIP Reduced LPS-Induced Migration and Inflammation in BV-2 Cells

**Figure 2:**
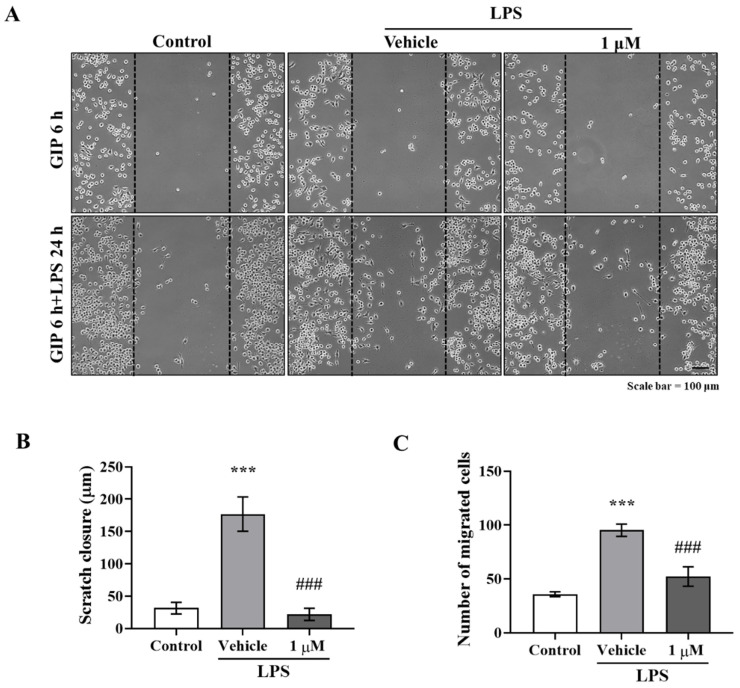

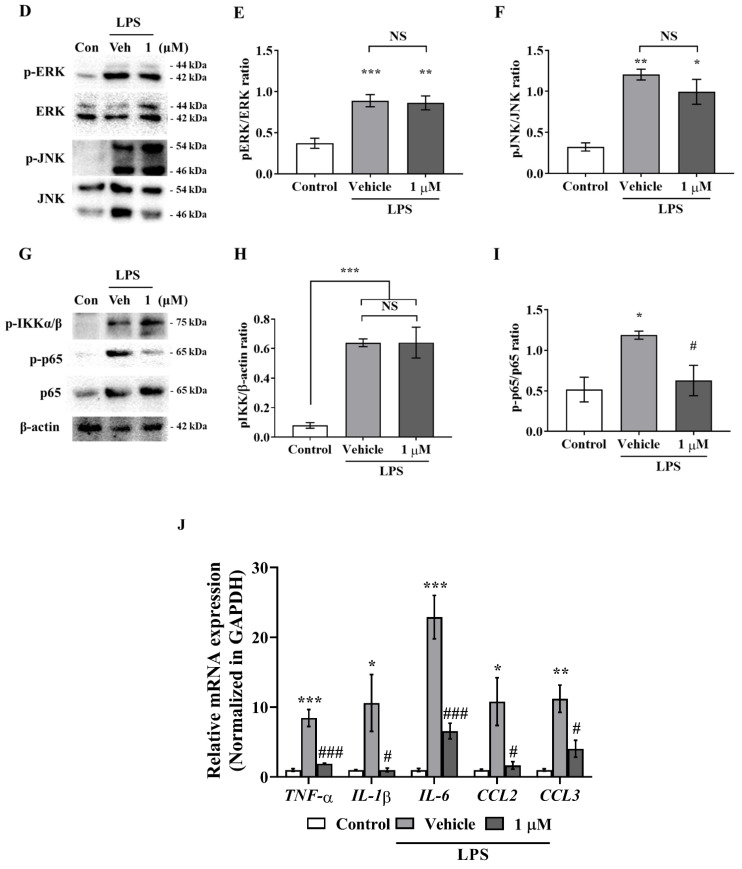
Anti-inflammatory effect of IAGIP on BV-2 cells. (**A**) Representative images showing that IAGIP inhibited the migration activity of BV-2 cells (a microglial cell line). Scale bar = 100 μm. (**B**) Scratch areas were measured. Values are means ± SEs (*n* = 4–5). ^***^ *p* < 0.001 vs. vehicle-treated controls and ^###^ *p* < 0.001 vs. LPS-treated controls. (**C**) The number of migrated cells was counted. Values are means ± SEs (*n* = 5–6). ^***^ *p* < 0.001 vs. vehicle-treated controls and ^###^ *p* < 0.001 vs. LPS-treated controls. (**D**,**G**) Western blotting confirmed that IAGIP reduced inflammatory response in BV-2 cells without influencing MAPK phosphorylation. (**E**,**F**,**H**,**I**) Densitometric analysis of Western blots. More than three independent experiments were performed (*n* = 3–5). ^***^ *p* < 0.001, ^**^ *p* < 0.01, and ^*^ *p* < 0.05 vs. vehicle-treated controls, and ^#^ *p* < 0.05 vs. LPS-treated controls. (**J**) RT-PCR showed that IAGIP dramatically reduced LPS-induced inflammatory cytokine and chemokine levels. Values are means ± SEs (*n* = 3–4). ^***^ *p* < 0.001, ^**^ *p* < 0.01, and ^*^ *p* < 0.05 vs. vehicle-treated controls, and ^###^ *p* < 0.001 and ^#^ *p* < 0.05 vs. LPS-treated controls.

Activated microglia typically migrate to lesions, and GSK-3β is closely related to microglia migration [21,22]. BV-2 murine microglial cells pre-treated with 1 μM of IAGIP for 6 h and then co-treated with 1 μg/mL of LPS for 24 h were subjected to migration assays. Representative images showed that LPS stimulated BV-2 cells to close scratches faster than controls and that this was significantly inhibited by IAGIP (Figure 2A–C). In addition, LPS induced the phosphorylations of ERK, JNK, IKK, and p65, which demonstrated that LPS activates the MAPK-NF-κB pathway in BV-2 cells (Figure 2D,G). Although IAGIP pre-treatment did not affect LPS-induced MAPK or IKK activation, it effectively suppressed the LPS-induced phosphorylation of p65 (Figure 2E,F,I) and significantly reduced the LPS-induced upregulations of inflammatory cytokines and chemokines (Figure 2J), which showed the anti-inflammatory effect of IAGIP in BV-2 cells involved blocking NF-κB signaling.

### 2.3. IAGIP Had No Neuroprotective Activity in Primary Neurons

**Figure 3:**
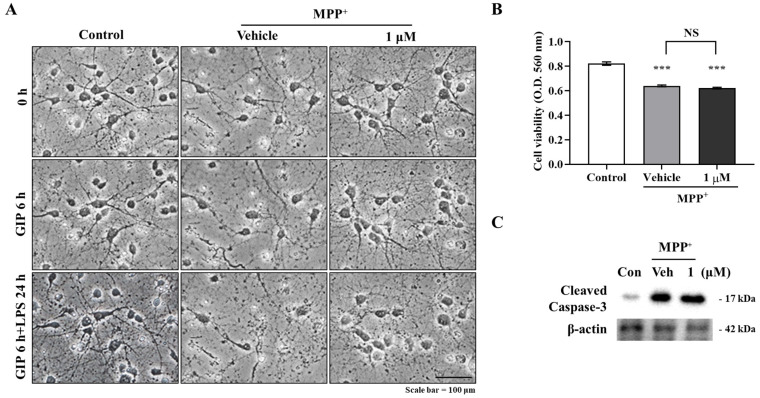
IAGIP had no neuroprotective effect on primary neurons. (**A**) Representative images showing that IAGIP did not protect primary neurons from MPP^+^-induced neuronal damage. Scale bar = 100 μm. (**B**) MTT assays confirmed that IAGIP did not protect neurons directly. Results are means ± SEs (*n* = 4). ^***^ *p* < 0.001 vs. vehicle-treated controls. (**C**) Western blotting showed that IAGIP did not reduce MPP^+^-induced caspase-3 cleavage.

To evaluate the neuroprotective effects of IAGIP, primary neurons were cultured, pre-treated with IAGIP for 6 h, and then co-treated with MPP^+^ for 24 h. IAGIP was not able to protect neurons against MPP^+^-induced neuronal death and did not block MPP^+^-induced caspase activation (Figure 3A–C). These findings indicate that IAGIP specifically reduced glial activation.

### 2.4. IAGIP Ameliorated MPTP-Induced Motor Deficits in Our Mouse Model of PD

**Figure 4:**
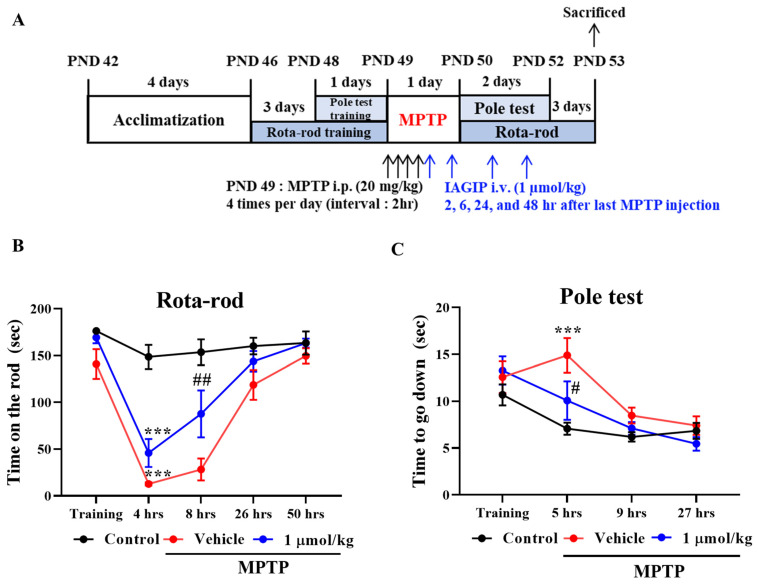
IAGIP suppressed MPTP-induced motor dysfunction in mice. (**A**) Design of the in vivo experiment. (**B**) Motor function was assessed using the Rota-rod test. Mice were pre-trained for 3 days to remain on the rod for 180 s. Tests were performed at 4, 8, 26, and 50 h after final MPTP injection at a rod speed of 30 rpm. Values are means ± SEs (*n* = 9–10 mice/group). ^***^ *p* < 0.001 vs. vehicle-treated controls and ^##^ *p* < 0.01 vs. MPTP-treated controls. (**C**) Bradykinesia was assessed using the pole test. Mice were pre-trained to descend the vertical pole, and tests were performed 5, 9, and 27 h after final MPTP injection. Values are means ± SEs (*n* = 8–9 mice/group). ^***^ *p* < 0.001 vs. vehicle-treated controls and ^#^ *p* < 0.05 vs. MPTP-treated controls.

The effects of IAGIP were also evaluated in an MPTP-induced mouse model of PD. Mice were pre-trained for three days for the Rota-rod test and for 1 day for the pole-test. Mice were intraperitoneally (i.p.) injected MPTP (20 mg/kg) 4 times at 2 h intervals to rapidly induce a PD-like pathology. Then, IAGIP was injected intravenously (i.v.) at 1 μmol/kg 2, 6, 24, and 48 h after last MPTP injection (Figure 4A). All MPTP-treated mice immediately fell off the rotating rod when tested 4 h after the last MPTP injection, indicating that motor function was impaired. Interestingly, motor function recovery was significantly faster for 1 μmol/kg IAGIP-treated mice than for MPTP-treated controls at 8 h after the last MPTP injection (Figure 4B). In addition, the pole test showed that MPTP-treated controls descended slowly and trembled, whereas MPTP/IAGIP-treated mice performed obviously better at 5 h after the last MPTP injection (Figure 4C).

### 2.5. IAGIP Suppressed Astroglial Activation and Neuroinflammation in the PD Mouse Model

**Figure 5:**
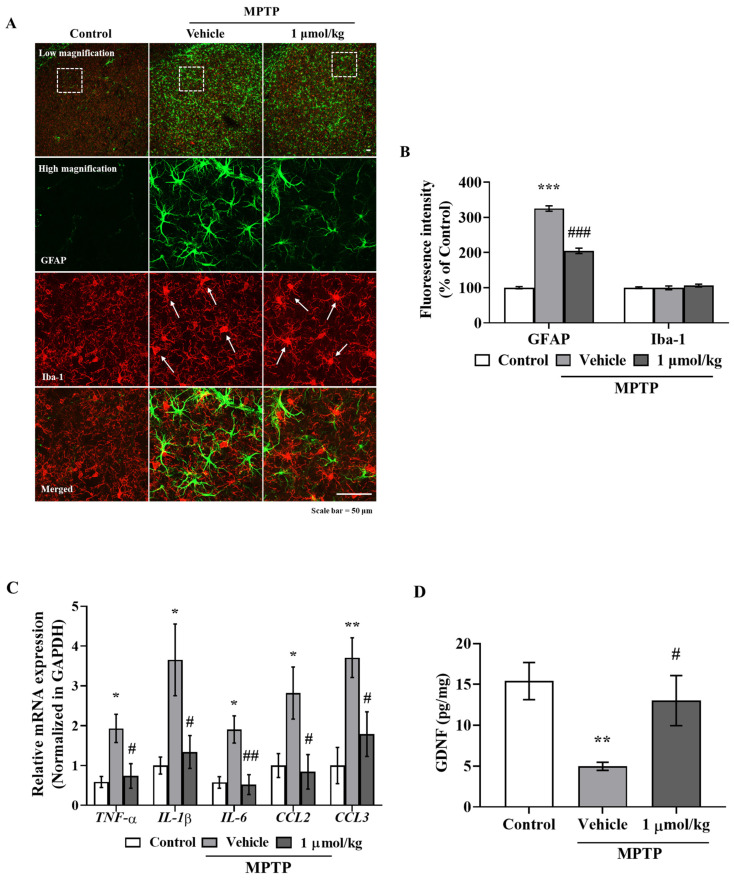
IAGIP reduced MPTP-induced astroglial activation in the striatum. (**A**) STR sections were double immunostained using GFAP (astrocyte marker) and Iba-1 (microglia marker) antibodies. Representative images in the top row showed the overall expression patterns of GFAP and Iba-1, and high magnification images were stacked z-sections. Scale bar = 50 μm. (**B**) Quantitative analysis of GFAP (green) and Iba-1 (red) fluorescence intensities. Results are presented as means ± SEs (*n* = 5–6 mice/group). ^***^ *p* < 0.001 vs. vehicle-treated controls and ^###^ *p* < 0.001 vs. MPTP-treated controls. (**C**) RT-PCR showed that IAGIP significantly reduced MPTP-induced inflammatory cytokine and chemokine levels in STR. Values are means ± SEs (*n* = 3–6 mice/group). ^**^ *p* < 0.01 and ^*^ *p* < 0.05 vs. vehicle-treated controls, and ^##^ *p* < 0.01 and ^#^ *p* < 0.05 vs. MPTP-treated controls. (**D**) GDNF levels in STR were assessed by ELISA. Values are means ± SEs (*n* = 5–6 mice/group). ^**^ *p* < 0.01 vs. vehicle-treated controls and ^#^ *p* < 0.05 vs. MPTP-treated controls.

To investigate the anti-inflammatory effects of IAGIP in our mouse model of PD, sections of striatum (STR) were double immunostained with GFAP and Iba-1 antibodies. Observations showed many GFAP-positive cells (activated astrocytes) in STR damaged by MPTP but few GFAP-positive astrocytes in normal controls. Interestingly, IAGIP treatment significantly reduced the number of activated astrocytes in striatal sections (Figure 5A). In STR, MPTP treatment caused microglial swelling (indicative of activation) (Figure 5A, white arrow) but IAGIP had no effect on this swelling (Figure 5A,B). RT-PCR showed that MPTP induced the expressions of inflammatory cytokines and chemokines in striatal homogenates and that IAGIP effectively suppressed these MPTP-induced mRNA expressions (Figure 5C). Additionally, ELISA assays showed that the glial-derived neurotrophic factor (GDNF) levels in STR were decreased by MPTP and that IAGIP treatment recovered the GDNF levels (Figure 5D). These findings showed that IAGIP effectively attenuated astroglial activation and neuroinflammation in our mouse model of PD.

### 2.6. IAGIP Protected Dopaminergic Neurons in the Nigrostriatal Pathway in PD Mouse Model

**Figure 6:**
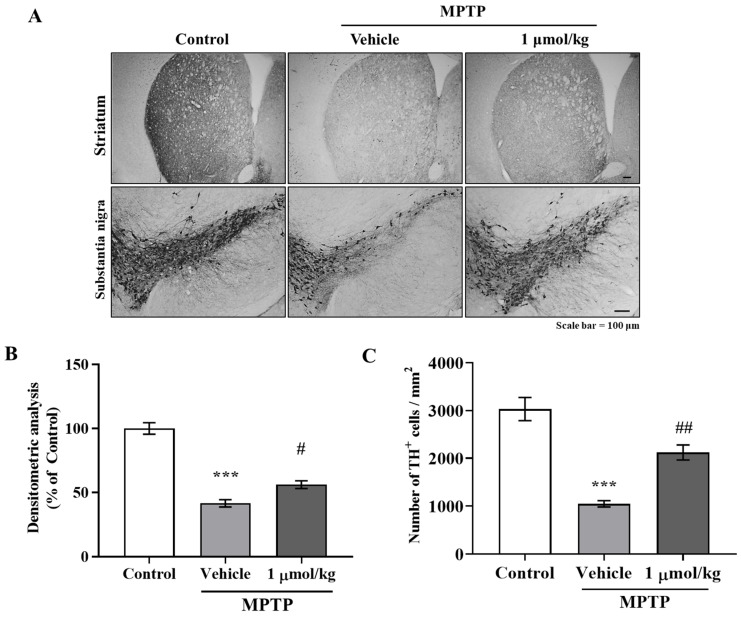
IAGIP prevented dopaminergic neuronal loss in the MPTP-induced mouse model of PD. (**A**) The neuroprotective effects of IAGIP on the nigrostriatal pathway were investigated immunohistochemically. Scale bar = 100 μm. (**B**) Densitometry was used to assess TH levels in STR. Values are means ± SEs (*n* = 5–6 mice/group). ^***^ *p* < 0.001 vs. vehicle-treated controls and ^#^ *p* < 0.05 vs. MPTP-treated controls. (**C**) TH-positive dopaminergic neurons in the SN were counted, and results are presented as means ± SEs (*n* = 5–6 mice/group). ^***^ *p* < 0.001 vs. vehicle-treated controls and ^##^ *p* < 0.01 vs. MPTP-treated controls.

To determine whether IAGIP had a protective effect on dopaminergic neurons in our PD mouse model, we visualized dopaminergic neurons in the nigrostriatal pathway (STR and substantia nigra (SN)) by performing tyrosine hydroxylase (TH) immunohistochemistry. Representative images showed that TH levels were significantly reduced by MPTP treatment in STR and SN and that IAGIP treatment effectively suppressed MPTP-induced dopaminergic neuronal damage and loss (Figure 6A). A densitometric analysis of STR and TH-positive cell counts in SN showed that IAGIP had neuroprotective effects in our mouse model (Figure 6B,C). These findings suggest that the anti-inflammatory effects of IAGIP might protect neurons in the nigrostriatal pathway against PD-associated neurodegeneration.

## 3. Discussion

Excessive activation of GSK-3β has negative effects in neurodegenerative diseases. For example, hyperactive GSK-3β promotes tau phosphorylation and amyloid beta deposition in AD [23] and facilitates neuronal death in MPP^+^, rotenone, or 6-hydroxydopamine-induced in vitro PD models [24,25]. Moreover, levels of GSK-3β phosphorylated at Ser9 were reported to be lower in MPTP-induced C57BL/6 mice, which indicated that GSK-3β is activated in PD lesions. In addition, MPTP-induced dopaminergic neuronal loss was prevented in GSK-3β conditional knockout mice, which indicates that GSK-3β activation is involved in MPTP-induced neurodegeneration [26]. These findings suggest that the inhibition of excessive GSK-3β activity provides a possible means of controlling neuroinflammation in neurodegenerative diseases.

Since GSK-3β has a crucial role in metabolism, insulin signaling, protein regulation, and inflammation, GSK-3β inhibition is considered an attractive target for therapeutic intervention in metabolic and neurodegenerative diseases [23]. However, the design of specific inhibitors of intracellular kinases including GSK-3β is very difficult because the kinase families share conserved ATP-binding sites, and thus, currently developed kinase inhibitors have mostly off-target effects [27]. In particular, since GSK-3β is a constitutively active kinase, excessive GSK-3β inhibition could have adverse effects by disrupting its physiological roles. For example, SB216763, a well-known GSK-3β inhibitor, was effective in attenuating Aβ-induced neurotoxicity in an AD model, but it induced neuronal death, gliosis, and behavioral deficits in control animals [28]. Therefore, it would be important to design a GSK-3β inhibitor to selectively inhibit the activity of the kinase when it is excessively activated in a pathological condition without affecting its physiological roles in normal condition.

IAGIP was designed to directly target the N-terminal region of GSK-3β, which includes Ser9, and to block GSK-3β activity [29]. To further enhance the GSK-3β inhibitory activity, the PPPSPxS motif of LRP6 (an essential co-receptor of Wnt) was conjugated to directly inhibit GSK-3β. Furthermore, the LRP6 motif was linked to an IKK-recognized motif (RHDSGLDSMKD) derived from IκB protein isoforms, and a serine residue was introduced between the two motifs [16]. Therefore, when IKK is activated in the inflammatory state, serine residues in the IKK-recognized motif are phosphorylated by IKK, and the two serine and threonine residues in the LRP6 motif are phosphorylated by GSK-3β and CK1. Although the LRP6 motif cannot inhibit GSK-3β in its non-phosphorylated state, when phosphorylated, it potently inhibits GSK-3β [16,30]. The beneficial effects of IAGIP have only been evaluated in a rat colitis model to date, and thus, this is the first study to investigate its effects in PD models.

Activated microglia and astrocytes are features of neuroinflammation, and chronically reactive glial cells can promote neurodegeneration [31]. Furthermore, it has been reported that the inhibition of GSK-3β alleviates neuroinflammation by reducing microglial migration and nitric oxide (NO) production [21]. As was expected, we found that IAGIP greatly reduced MPP^+^-induced GFAP elevation and LPS-stimulated BV-2 migration (Figure 1A–C and Figure 2A–C), and interestingly, although IAGIP did not influence stimulus-mediated MAPK phosphorylation and IKK activity in primary astrocyte and BV-2 cells, it significantly attenuated p65 phosphorylation (Figure 1D,G, and Figure 2D,G). These findings suggest that the IAGIP-mediated inhibition of p65 is independent of the activations of MAPKs or IKK. In particular, this independence of IKK might be important for the inhibitory activity of IAGIP as its IKK-recognized motif should be phosphorylated by active IKK. In a previous study, lithium chloride (LiCl) did not affect IκBα degradation, IKK activity, or NF-κB binding activity but decreased the NF-κB-dependent inducible nitric oxide synthase gene expression in TNF-α induced rat hepatocytes. This probably occurred because the p65 sequence contains four potential GSK-3 phosphorylation sites within its COOH-terminal transactivation domains [32]. Another possibility is that GSK-3β directly regulates the phosphorylation of NF-κB essential modulator (NEMO) (an IKK complex subunit), and thus, the inhibition of GSK-3β blocks IκBα phosphorylation by inhibiting NEMO, thereby preventing the nuclear translocation of p65 [33].

Although GSK-3β has been regarded as a regulator of inflammation for more than ten years, its specific role in inflammation remains unclear [14]. Nevertheless, numerous studies have demonstrated that inhibition of GSK-3β reduces inflammatory response; for example, SB216763 (a GSK-3β inhibitor) reduced LPS-stimulated IL-6 levels in porcine adipocytes [34]. In brain, GSK-3β promotes STAT3 (signal transducer and activator of transcription-3) phosphorylation, and thus, knockdown of GSK-3β significantly inhibited IL-6 levels in LPS-injected mouse brain and LPS-treated primary glial cells [35]. Green and Nolan demonstrated that SB216763 or LiCl dramatically reduced LPS-stimulated IL-1β, TNF-α, and IL-10 levels in mixed cortical glial cell cultures [36]. In addition, GSK-3 inhibition reduced LPS-mediated inflammatory mediator levels such as NO, glutamate, TNF-α, and IL-6 in BV-2 cells [22]. In the present study, MPP^+^ and LPS induced proinflammatory cytokines, and these inductions were greatly suppressed by IAGIP in primary astrocytes and BV-2 cells, respectively (Figure 1K and Figure 2J). These results indicate that the inhibition of GSK-3β plays an important role in proinflammatory cytokine suppression by inhibiting NF-κB activation in glial cells.

The anti-inflammatory effects of IAGIP effectively blocked nigrostriatal degeneration in our MPTP-induced mouse model of PD. The MPTP-induced mouse model has several limitations. First, the most common drawback of this model is that it rarely forms Lewy bodies. Second, the MPTP-induced PD model is relatively transient, which differs from sustained progression in PD patients. Third, most rodent models are mainly used in adolescence even though PD occurs most frequently in older patients [37,38]. Nevertheless, the MPTP model readily develops the pathology of PD and has fewer ethical considerations than those of other animal models such as nonhuman primate. It is also useful for understanding mitochondrial dysfunction and autophagy in PD, and the MPTP mouse model closely mimics the parkinsonian symptoms including non-motor symptoms in human PD [39]. Rota-rod testing showed IAGIP significantly reduced MPTP-induced motor dysfunction one day after last MPTP injection (Figure 4). Interestingly, IAGIP also effectively improved pole test motor performance. This pole test has been used to evaluate motor dysfunction in bradykinesia (a representative symptom of PD) [40], which suggests that IAGIP might effectively attenuate bradykinesia. Furthermore, IAGIP effectively lowered astrocyte activation but not microglial activation in the STR of MPTP-treated PD mice, which we suspect was probably because mice were sacrificed at 3 days after MPTP administration for behavioral tests. Microglia are rapidly activated by stimuli to release inflammatory cytokines and to recruit astrocytes, and these activities persist for several hours to a day. Astrocytes begin to activate about two or three days after damage occurs [41,42], and thus, the suppressive effect of IAGIP on microglial activation may not have been observed given the experimental timings we adopted. Interestingly, in STR, proinflammatory cytokines were increased by MPTP and effectively decreased by subsequent IAGIP treatment (Figure 5C). In addition, GDNF (glial-derived neurotrophic factor) levels were significantly reduced in MPTP-administered mice. Astrocytes are activated into two types with opposing roles, called ‘A1’ and ‘A2’. A1-type astrocytes are neurotoxic, upregulate inflammatory cytokines, and cause neuronal damage, whereas A2 astrocytes release neurotrophic factors that promote synapse repair [41,43], which suggests that neurodegenerative A1-type astrocyte activation was triggered in MPTP-induced PD mice and that IAGIP blocked this activation. Additionally, we found that these anti-inflammatory effects of IAGIP effectively attenuated dopaminergic neuronal loss and damage in our PD mouse model. These findings suggest that the anti-inflammatory effect of IAGIP might beneficially protect against nigrostriatal neurodegeneration in PD.

In conclusion, we found that the anti-inflammatory activity of IAGIP is only evident under inflammatory conditions in PD. Moreover, our findings suggest that targeting glial activation and neuroinflammation might be an effective means of protecting dopaminergic neurons in PD and that IAGIP has potential therapeutic use in PD.

## 4. Materials and Methods

### 4.1. Materials

Peptides (>95% purity) were obtained from Peptron Inc. (Daejeon, Korea). To improve cell permeability, a cell-permeable sequence (YGRRARRRARR) was attached to the N terminal of the IKK-activated GSK3β inhibitory peptide (IAGIP; EPVPPPPTPRSSRHDSGLDSMKD). Chloroform, gentamycin, isopropanol, MPTP (1-methyl-4-phenyl-1,2,3,6-tetrahydropyridine), MPP^+^ (1-methyl-4-phenylpyridium), MTT (3-[4,5-dimethyl-2-thiazolyl]-2,5-diphenyl-2H-tetrazolium bromide), paraformaldehyde, and poly-L-lysine were obtained from Sigma-Aldrich (St. Louis, MO, USA). Alexa Fluor 488 and Alexa Fluor 568 were from Invitrogen (Waltham, MA, USA), and the Western blot detection reagent (ECL solution) was purchased from Advansta (Menlo Park, CA, USA).

### 4.2. Primary Astrocyte Culture

Primary astrocytes were isolated from the cerebral cortices of Sprague Dawley (SD) rats on postnatal days (PND) 1–2 (Daehan Biolink Co. Ltd., Chungbuk, Korea). Briefly, cortices were dissected and chopped in ice-cold Hanks’ balanced salt solution (HBSS) buffer (Welgene Inc., Daegu, Korea). To isolate cells from debris, tissues were then treated with 0.25% trypsin (Welgene Inc., Daegu, Korea) for 30 min at 37 °C, washed with HBSS, mechanically dissociated, and plated in Dulbecco’s modified Eagle’s medium/nutrient mixture F-12 (DMEM/F12; Gibco, Waltham, MA, USA) medium containing 10% fetal bovine serum (FBS; Welgene, Daegu, Korea) on poly-L-lysine coated plates. Cultures were maintained under the same conditions and used for experiments after 14–18 days in vitro.

### 4.3. Immunocytochemistry

Primary astrocytes were seeded in 35 mm poly-L-lysine-coated plastic culture dishes, pre-treated with 1 μM IAGIP for 6 h, treated with 200 μM MPP^+^ for 24 h, washed with warm phosphate-buffered saline (PBS), fixed with 4% paraformaldehyde (PFA) in PBS (pH 7.4) for 20 min at 37 °C, and blocked with TBS-TS (tris buffered saline/0.1% Triton X-100/3% goat serum) for 30 min at room temperature. Cells were then incubated with primary antibodies for GFAP (mouse monoclonal; Cell Signaling Technology, Danvers, MA, USA) at 4 °C overnight, washed with TBS-TS and then TBS, incubated with anti-mouse IgG labeled with Alexa Fluor 488 (Invitrogen) for 3 h at room temperature, and washed with TBS. Images were acquired using a FV10i FLOU-View confocal microscope (Olympus; Tokyo, Japan).

### 4.4. Western Blot Analysis

After treatments, cells or tissues were subjected to SDS-PAGE (sodium dodecyl sulfate-polyacrylamide gel electrophoresis), and protein concentrations in supernatants were measured using a Bio-Rad (Hercules, CA, USA) protein assay kit and bovine serum albumin (BSA) as the standard. Proteins in supernatants (17 μg protein per lane) were then separated by 12% SDS-PAGE and transferred to Immobilon-P^SQ^ membranes (Millipore; MA, USA). Membranes were immediately placed in 5% skim milk for 30 min and then incubated with the following primary antibodies; GFAP (mouse monoclonal; Cell Signaling Technology), cleaved caspase-3, caspase-3, p-ERK, ERK, p-IKKα/β, p-JNK, JNK, p-p65, and p65 (rabbit polyclonal; Cell Signaling Technology), and β-actin (mouse monoclonal; Santa Cruz Biotechnology, Dallas, CA, USA) in Tris-HCl-based buffer containing 0.2% Tween 20 (TBS-T; pH 7.5) overnight at 4 °C. The membranes were then washed and incubated with secondary monoclonal anti-mouse and polyclonal anti-rabbit antibodies (1:10,000; Santa Cruz Biotechnology) in TBS-T for 2 h. Horseradish peroxidase conjugated secondary antibody labeling was detected by enhanced chemiluminescence (ECL) using a cooled CCD camera system (ATTO Ez-Capture II; Atto Corp., Tokyo, Japan). Relative protein levels were quantified by densitometry with respect to total forms (ERK, JNK, or p65) or β-actin as the loading control.

### 4.5. Luciferase Assay

Primary astrocytes were seeded in 96-well plate (1 × 10^6^ cells/mL) in serum-free medium and transfected with 0.1 μg of 4xNF-κB plasmid (Addgene; Watertown, MA, USA) for 24 h using ViaFect^TM^ transfection reagent (Promega; Madison, WI, USA) in Opti-MEM (Gibco). Cells were then pre-treated with 1 μM IAGIP for 6 h, treated with 200 μM MPP^+^ for 6 h, washed with PBS, and lysed using the ONE-Glo^TM^ Luciferase Assay System (Promega; Madison, WI, USA). The NF-κB-dependent activity of luciferase reporter was measured using a GENious luminometer (TECAN, Salzburg, Austria).

### 4.6. Real-Time Polymerase Chain Reactions

Cells and tissues were homogenized using RiboEX^TM^ reagent (GeneAll; Seoul, Korea), chloroform was added, and the solution was shaken vigorously for 15 min. The aqueous phase was then transferred to fresh tubes, isopropanol was added, and the solution was incubated for 15 min at 4 °C and centrifuged for 15 min at 12,000× *g*. The supernatants were removed, and the pellets were washed with 75% ethanol and centrifuged for 5 min at 8000× *g*. The RNA pellets obtained were dried and dissolved in diethylpyrocarbonate water, and the mRNA concentrations were calculated. mRNA was reverse transcribed to cDNA using SuPrimeCript RT Premix (Genetbio Inc.; Daejeon, Korea). Real-time PCR analysis was performed using SYBR green master mix (BIOLINE, London, UK) and the CFX Connect System (Bio-Rad Inc.).

### 4.7. Cell Line Culture

BV-2 cells (a murine microglial cell line) were obtained from ATCC (Manassas, VA, USA) and grown in Dulbecco’s modified Eagle’s medium (DMEM) supplemented with 10% FBS and 1% penicillin/streptomycin in a humidified 5% CO_2_/95% air atmosphere at 37 °C. The medium was replaced at 2–3 days intervals, and the cells were seeded in cell culture plates for experiments.

### 4.8. Migration Assay

BV-2 cells were seeded in 6-well plates in growth medium and incubated at 37 °C in a 5% CO_2_ in an air atmosphere to required densities until 50% confluent. The cells were then pre-treated with IAGIP (1 μM) for 6 h, and the center of the monolayers in each well were scratched using a sterile yellow pipette tip. The cells were then washed twice with PBS to remove detached cells, immediately treated with 1 μg/mL LPS, and observed for 24 h. The images were taken immediately after being scratched and 24 h after LPS treatment using a Nikon ECLIPSE TE 2000-U microscope (Nikon, Tokyo, Japan). Three independent experiments were performed, and similar results were obtained. Scratch areas were measured using NIS Elements BR Ver. 4.00.12 (Nikon, Tokyo, Japan).

### 4.9. Primary Neuron Culture

Primary neuron cultures were performed as previously described. Briefly, cortical tissues from embryonic days 18–19 of SD rats were excised in ice-cold Hanks’ balanced salt solution (HBSS) buffer (Welgene Inc., Daegu, Korea) containing 0.1 mg/mL gentamycin and were incubated in 2 mg/mL of trypsin for 15 min. The tissues were dissociated by trituration, and the cells obtained were plated on poly-L-lysine coated culture dishes in neurobasal medium (Gibco) supplemented with 2% B-27 (Gibco), 2 mM L-glutamine, and 25 μM glutamate for 24 h and then transferred to glutamate-free neurobasal medium. Experiments were performed 7 days after plating when most neurons had matured.

### 4.10. MTT Assay

Primary neurons (5 × 10^5^ cells/mL) were seeded in 96-well plates, cultured for 7 days, pre-treated with 1 μM IAGIP for 6 h, and co-treated with 500 μM MPP^+^ for 24 h. The medium was then removed, and 200 μL of a 0.5 mg/mL MTT solution in PBS was added to each well. The cells were then incubated at 37 °C for 4 h, the MTT solution was removed, and the cells were lysed in DMSO–ethanol (1:1). The amounts of formazan produced were quantified by measuring absorbance using a Multiskan FC microplate reader at 560 nm (Thermo Fisher Scientific, Waltham, MA, USA).

### 4.11. Animal Housing and Treatment

Male C57BL/6 mice (6 weeks old, 20–23 g) were obtained from Daehan Biolink Co. Ltd. (Chungbuk, Korea). The animals were maintained under temperature- and light-controlled conditions (20–23 °C under a 12 h light cycle) and were provided food and water *ad libitum*. The animals were randomly allocated to three groups of 10 to 12 animals: to a vehicle control group, an MPTP control group, or an MPTP + 1 μmol/kg IAGIP group. All animals were acclimatized for 1 week before drug administration. MPTP was injected i.p. at 20 mg/kg four times at 2 h intervals to induce acute PD symptoms, and IAGIP (1 μmol/kg dissolved in PBS) was injected i.v. four times at 2, 6, 24, and 48 h after the last MPTP injection. A schematic of the in vivo experiments is provided in Figure 4A. The animal protocol used in this study was reviewed and approved beforehand by the Institutional Animal Care Committee of Pusan National University (PNU-IACUC; Approval Number PNU-2021-2903).

### 4.12. Rota-Rod Test

All mice were pre-trained for 3 days to maintain themselves on the rod for 180 s. The training sessions involved four consecutive runs at a rod speed of 30 rpm, and mice were tested 4, 8, 26, and 50 h after final MPTP administration (2 h after each IAGIP injection) 4 times at a rod speed of 30 rpm. The mice were sacrificed for histological and biological analyses 72 h after completing Rota-rod tests.

### 4.13. Pole Test

The pole test was used to assess balance and bradykinesia and was performed 1 h after Rota-rod testing. A vertical wooden pole (50 cm high/1 cm diameter) wrapped with medical gauze was placed in a home cage, a mouse was placed heads up at the top of the pole, and recording started when the mouse began to turn to descend to the cage floor. The test was repeated 3 times per animal. When a mouse fell off the pole immediately due to motor dysfunction, a default score of 30 s was assigned.

### 4.14. Tissue Preparation

For histological studies, mice were anesthetized with diethyl ether, perfused intracardially with 0.9% NaCl in 0.1 M PBS (pH 7.4), and fixed with 4% PFA in 0.1 M PBS. Brains were then removed, placed in the same fixative solution at 4 °C overnight, and transferred to 30% sucrose. Cryoprotected brains were serially sectioned at 40 μm in the coronal plane using a MICROM HM430 freezing microtome (MICROM; Walldorf, Germany), and sections were stored at 4 °C in Dulbecco’s phosphate-buffered saline (DPBS) solution containing 0.1% sodium azide.

### 4.15. Double Fluorescence Immunohistochemistry

Brain sections were blocked with TBS-TS for 30 min and incubated with primary antibodies (anti-GFAP and anti-Iba-1 rabbit polyclonal (Wako, Tokyo, Japan)) in TBS-TS overnight at 4 °C. Sections were then incubated with secondary anti-mouse IgG labeled with Alexa Fluor 488 and anti-rabbit IgG labeled with Alexa Fluor 568 for 3 h at room temperature, washed with TBS, and mounted onto slides using aqueous and dry mounting medium (Biomeda Corp., Foster City, CA, USA). Images were acquired using a ZEISS LSM800 confocal microscope (ZEISS, Oberkochen, Germany).

### 4.16. ELISA Assay

GDNF protein levels were quantified using a GDNF Emax Immunoassay System (Promega; Madison, WI, USA) according to the manufacturer’s instructions. Briefly, striatal homogenates were added to pre-coated 96-well plates containing anti-GDNF monoclonal antibody and shaken for 6 h at room temperature. The plates were then washed with TBS-T and treated with anti-human GDNF polyclonal antibody overnight at 4 °C. The next day, the plates were washed with TBS-T and treated with HRP-conjugated anti-chicken IgY for 2 h at room temperature and then with TMB solution for 15 min without shaking. The reaction was stopped by adding 1 N HCl, which caused a color change from blue to yellow. Absorbances were measured at 450 nm using a Multiskan FC microplate reader (Thermo Fisher Scientific).

### 4.17. Diaminobenzidine Immunohistochemistry

Briefly, to block endogenous peroxidase activity, brain sections were treated with 0.6% H_2_O_2_ in Tris-buffered saline (TBS; pH 7.5), blocked in TBS-TS for 30 min, and incubated with primary anti-TH antibody (mouse monoclonal; Chemicon, Rolling Meadows, IL, USA) in TBS-TS at 4 °C. The sections were then exposed to appropriate biotinylated secondary goat anti-mouse IgG antibodies (Vector Laboratories, San Francisco, CA, USA) at room temperature for 3 h, incubated in ABC solution (ABC reagent Elite Kit, Vector Laboratories) at room temperature for 1 h, and developed using diaminobenzidine (DAB) solution. Images were obtained using a Nikon ECLIPSE TE 2000-U microscope (Nikon, Tokyo, Japan). Densitometric analysis of TH expression intensity in STR (five to six sections per mouse) were performed using a FluorChem SP (Alpha Innotech, San Leandro, CA, USA), and the numbers of TH positive dopaminergic neurons in SN were counted.

### 4.18. Statistical Analysis

The significances of intergroup differences were determined using the *t*-test or analysis of variance (ANOVA) followed by Tukey’s multiple comparison test in Prism Ver. 8.0. (GraphPad Software Inc., San Diego, CA, USA). *p* values of <0.05 were considered statistically significant.

## Data Availability

The data that support the findings of this study are available from the corresponding author upon reasonable request.
